# A novel quality assurance procedure for trajectory log validation using phantom‐less real‐time latency corrected EPID images

**DOI:** 10.1002/acm2.13202

**Published:** 2021-02-26

**Authors:** Seng Boh Lim, Benjamin J. Zwan, Danny Lee, Peter B. Greer, Dale Michael Lovelock

**Affiliations:** ^1^ Department of Medical Physics Memorial Sloan Kettering Cancer Center New York NY USA; ^2^ School of Mathematical and Physical Sciences University of Newcastle Newcastle NSW Australia; ^3^ Central Coast Cancer Centre Gosford Hospital Gosford NSW Australia; ^4^ Department of Radiation Oncology Allegheny Health Network Pittsburgh PA USA; ^5^ Department of Radiation Oncology Calvary Mater Hospital Newcastle Waratah NSW Australia

**Keywords:** EPID, log file analysis, patient‐specific QA, quality assurance

## Abstract

The use of trajectory log files for routine patient quality assurance is gaining acceptance. Such use requires the validation of the trajectory log itself. However, the accurate localization of a multileaf collimator (MLC) leaf while it is in motion remains a challenging task. We propose an efficient phantom‐less technique using the EPID to verify the dynamic MLC positions with high accuracy. Measurements were made on four Varian TrueBeams equipped with M120 MLCs. Two machines were equipped with the S1000 EPID; two were equipped with the S1200 EPID. All EPIDs were geometrically corrected prior to measurements. Dosimetry mode EPID measurements were captured by a frame grabber card directly linked to the linac. All leaf position measurements were corrected both temporally and geometrically. The readout latency of each panel, as a function of pixel row, was determined using a 40 × 1.0 cm^2^ sliding window (SW) field moving at 2.5 cm/s orthogonal to the row readout direction. The latency of each panel type was determined by averaging the results of two panels of the same type. Geometric correction was achieved by computing leaf positions with respect to the projected isocenter position as a function of gantry angle. This was determined by averaging the central axis position of fields at two collimator positions of 90° and 270°. The radiological to physical leaf end position was determined by comparison of the measured gap with that determined using a feeler gauge. The radiological to physical leaf position difference was found to be 0.1 mm. With geometric and latency correction, the proposed method was found to be improve the ability to detect dynamic MLC positions from 1.0 to 0.2 mm for all leaves. Latency and panel residual geometric error correction improve EPID‐based MLC position measurement. These improvements provide for the first time a trajectory log QA procedure.

## 1 INTRODUCTION

Trajectory log files are records automatically generated at the end of each field delivered in treatment mode by a radiotherapy machine. These log files are a sequence of snap shots of the machine state taken at fixed intervals. The interval depends on the machine type, being as short as 20 ms for new models. The machine state record includes parameters, such as energy, dose rate, position of each multileaf collimator (MLC) leaf, gantry angle, collimator angle, and couch position. Because of their availability, correlation with the actual treatment delivery^1^, ^2^ and high precision, the use of trajectory log files is gaining popularity as a QA tool for patient specific and routine QA.^1^, ^2^, ^3^, ^4^, ^5^, ^6^, ^7^ Note, however, that the parameters recorded are taken from the machine's control system; they are not measurements and can be in error. MLC positions reported in the log have been found to deviate from the actual delivered positions.^1^ It is essential, therefore, to have a mechanism to both commission and to periodically QA the log file.^2^

Eckhause^2^ proposed a phantom‐based methodology to verify trajectory logs using the electronic portal imaging device (EPID). With the fiducials on the phantom used as landmarks, it was shown that it was possible to triangulate the leaf positions accurately enough to verify the positions reported in the log file. As this technique requires a phantom, the EPID was placed at source to detector distance (SDD) of 150 cm with a stationary gantry. This not only limits the verification of MLC within ±13 cm from isocenter for aS1000 panel but also limits the ability to check rotational delivery techniques such as VMAT.

Recently, Zwan et al^8^, ^9^ proposed an EPID‐based phantom‐less methodology for MLC QA. This allowed the EPID to be extended to the isocenter (SDD 100 cm) to image all the MLC leaves and to localize their position to within ±1.0 mm, which is sufficient to satisfy the tolerance required by TG‐142.^10^ Their approach was novel in that instead of using integrated images, the positions of the MLC leaves were determined while they were in motion using EPID images acquired at approximately 10 Hz. An accurate localization of moving leaves would make possible the verification of the MLC positions reported in the trajectory log. However, the accuracy is insufficient to be used to validate trajectory logs.

A factor contributing to the uncertainty is that the moment during the dose delivery at which a moving leaf in imaged was not corrected for timing delays or latencies in the readout of the EPID image. Because the leaf is moving, this results in a shift of the imaged position.

In this study, we describe a phantom‐less method to improve the accuracy leaf localization by correcting for EPID readout timing latencies.^11^ EPID positioning errors that depend on gantry angle, and MLC centerline calibration errors are also corrected for, resulting in a QA procedure with accuracy sufficient to QA trajectory log analysis. Such a QA process would increase efficiency and reduce the workload of clinical physicists.

## 2 MATERIALS AND METHODS

To validate a trajectory log, it is necessary to acquire EPID images of the leaves while they are in motion. Images were acquired in cine mode at approximately 10 frame per second (fps) via iTool Capture [Varian Medical Systems, Palo Alto, CA, USA]. The leaf positions were determined from the images and compared with the positions recorded in the trajectory log. This information is included in the header information of each image.

**FIG. 1:**
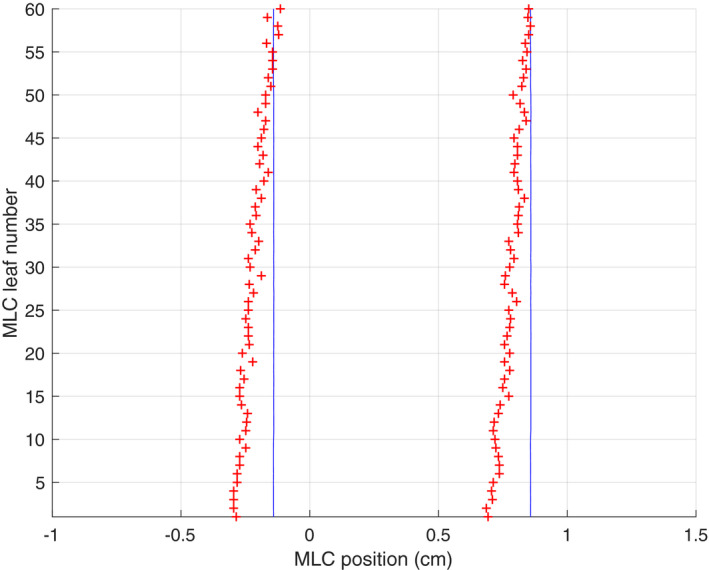
A snapshot of a SW slit delivery that shows the false positive error of the uncorrected EPID caused by EPID readout latency.

In EPID images of a sliding window (SW) slit field moving uniformly from right to left across the EPID panel, the slit appears slightly skewed with the leaf closest to the gantry appearing to be trailing the leaf furthest from the gantry by approximately 2 mm (Fig. 1).

It is hypothesized that the skewness is caused by the multiplexed readout of the EPID panel. The readout is row‐wise, with the row closest to the gantry being readout first.^11^ The time elapses between the imaging and readout of the last leaf pair (row 1) and that of the first leaf pair. The last leaf pair having moved on, is imaged and readout at an earlier position. Accurate determination of the positions of moving leaves requires this panel temporal delay, *t_d_*, to be corrected for.

All measurements were made on TrueBeam linacs [Varian Medical Systems, Palo Alto, CA, USA] equipped with Millennium^TM^ 120 multileaf collimators (M120 MLC). Prior to all measurements, EPIDs in this study were first calibrated using vendor's radiation isocenter calibration, IsoCAL, to optimize the panel offset^12^, ^13^ with gantry and collimator angles to within 0.5 mm accuracy at SDD 150 cm. All images were captured in dosimetry mode using a frame grabber [Matrox Imaging, Montreal, Canada] connected to the treatment units at a rate of 9.57 and 13.20 frames per second for the aS1000 and aS1200 panels, respectively, using iTools Capture software at SDD 100 cm. Since the flex map is a function of SDD,^14^ the residual EPID position errors resulting from the SDD change were evaluated on machines R444 and R445, both TrueBeam type Linacs, equipped with aS1200 and aS1000 EPID, respectively. To maximize the accuracy of the MLC detection, both *t_d_*, and geometric residual EPID position errors were accounted for in this study.

**FIG. 2:**
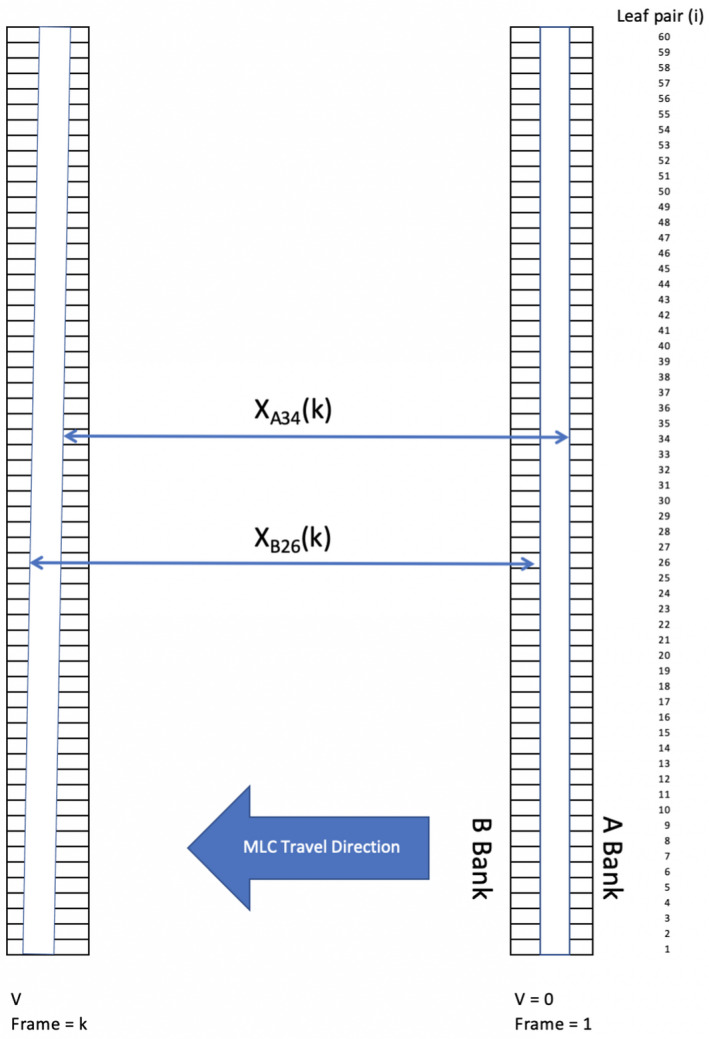
The distance traveled by each MLC of a SW field in frame k relative to the stationary position in frame 1 moving.

To determine *t_d_,* a 40.0 × 1.0 cm^2^ SW strip was first held stationary and then driven at a known constant speed, *v*, across 12.0 cm (Fig. 2). During leaf motion, approximately 120 to 1100 frames were acquired for MLC travel speeds of 2.5 cm/s and 0.3 cm/s, respectively. The travel time of any leaf pair *j* in the kth frame relative to the stationary position was determined by dividing the measuring the leaf displacements from the corresponding stationary position with the known MLC travel speed. The temporal delay error, *t_dj_*, of a leaf pair, *j*, at any instance *t* was defined as the time difference between the leaf pair *j* and a reference leaf pair *l*. For simplicity, leaf pair number 60 was taken as the reference to obtain *t_dj_* in this part of the study. On a Truebeam linac with the collimator at 0˚ rotation, leaf motion is in the transverse direction with leaf pair 1 most distant from the gantry stand. This *t_d_* should be constant. To test the hypothesis of *t_d_*, the MLC was first driven at 2.50 cm/s in right to left direction (R2L), 2.50 cm/s in left to right direction (L2R), and at 1.25 cm/s in R2L on an aS1200 panel. The *t_d_* of the both types of amorphous silicon EPIDs, the Varian aS1000 and aS1200, were determined by running the SW strip at the highest recommended leaf speed, *v* = 2.5 cm/s in R2L to minimize the measurement error. Each panel specific *t_d_* was determined by the measurements of two machines. A profile with 7 pixels width was extracted at the middle of each MLC leaf position. The maximum of the profile was determined by spline interpolation around the three highest pixel values. A sequence of approximately 1000 frames was acquired with the gap first stationary, then moving across the field at a velocity of 2.5 cm/s projected to isocenter. The positions of each leaf pair *j*, X_Aj_ (*k*) and X_Bj_ (*k*), of frame k traveling of speed of *v* were determined as the maximum gradient points of the penumbrae defined by the 80% and 20% of the maximum value. The dynamic displacements of each leaf pair, *x*_Aj_(*k*) and *x*_Bj_(*k*), were defined as the difference of the instantaneous leaf positions, X_Aj_ (*k*) and X_Bj_ (*k*), and the corresponding stationary positions, X_Aj_|*_v_*_=0_ and X_Bj_|*_v_*_=0_ (Fig. 2):

(1) $$\begin{array}{c} x_{\mathrm{Aj}}\left(k\right)=X_{\mathrm{Aj}}\left(k\right)‐X_{\mathrm{Aj}}{|}_{\begin{array}{c} v=0 \end{array}}\\ x_{\mathrm{Bj}}\left(k\right)=X_{\mathrm{Bj}}\left(k\right)‐X_{\mathrm{Bj}}{|}_{v=0} \end{array}$$

The mean center position of the leaf pair, *j*, is defined as:

(2) $$<x_{j}\left(k\right)>=<x_{\mathrm{Aj}}\left(k\right)+x_{\mathrm{Bj}}\left(k\right)>$$

The time delay, *t_d,j_*, of panel of a leaf pair *j* relative to a reference leaf pair *r* can be determined by:

(3) $$t_{d,\;j}=\frac{<x_{j}\left(k\right)‐x_{r}\left(k\right)>}{v}$$

The relationship between *t_d,j_* and position, *y*, perpendicular to the leaf travel direction from leaf *j* to *l* can also be expressed as:

(4) $$t_{d,\;j}=f\left(y\right)$$

And was derived with the linear regression. Here, leaf pair number 60, *r* = 60, closest to the first line readout was first used to determine the *t_d,j_* relationship for both panels. All the frames captured during the SW measurements were used to determine the average of each *t_dj_*. The images, which were acquired through a service port of a TrueBeam using iTools, are in a propriety XIM format, that in addition to the image, also contain header information recording a snapshot of the machine state corresponding to the middle of the integration interval of the middle row of the EPID. This snapshot interpolates the snapshots recorded in the trajectory log. During the time delay correction of all the measurements in this study, the center of the image was chosen to be the reference point tied to the timestamp, *t,* in the header of a particular frame. Relative to this point, the dynamic MLC position of each leaf *j* at *t*, was corrected by time shifting the MLC position at time, *t + t_dj_*.

An additional correction arises because of possible variation with gantry angle of the EPID position from its ideal position, where the projection of the radiation isocenter fall in the center of the panel. The panel residual errors relative to the radiation isocenter at each gantry angle were determined by two 360° arc deliveries with fixed MLC apertures of 2x2 cm^2^ and collimator angles set at 90° and 270° for each arc. The centers of the aperture at the collimator angles 90 and 270, C(x(θ), y(θ))_90_ and C(x(θ), y(θ))_270_, were measured as a function of the gantry angle θ. The projection of the radiation isocenter on the panel as a function of gantry angle was taken to be the mean position:

(5) $$C{\left(x\left(\theta \right),y\left(\theta \right)\right)}_{\mathrm{ISO}}=\frac{\left[C{\left(x\left(\theta \right),y\left(\theta \right)\right)}_{90}+C{\left(x\left(\theta \right),y\left(\theta \right)\right)}_{270}\right]}{2}$$

Combining (4) and (5), the MLC positions at any gantry angle and time were determined. The deviation of MLC position of *j*th leaf pair, $\Delta x_{\mathrm{Aj}}\left(t,\theta \right)$ and $\Delta x_{\mathrm{Bj}}\left(t,\theta \right)$, from the reference positions, $x_{Aj,ref}\left(t\right)$ and $x_{Bj,ref}\left(t\right)$, at the gantry angle θ at time *t* relative to the radiation isocenter.

(6) $$\begin{array}{cc} \Delta x_{\mathrm{Aj}}\left(t,\theta \right){|}_{\mathrm{ref}} & =x_{\mathrm{Aj}}\left(t+t_{d,j}\right)‐x\left(\theta \right)‐x_{Aj,ref}\left(t\right)\\ \Delta x_{\mathrm{Bj}}\left(t,\theta \right){|}_{\mathrm{ref}} & =x_{\mathrm{Bj}}\left(t+t_{d,j}\right)‐x\left(\theta \right)‐x_{Bj,ref}\left(t\right) \end{array}$$

The relationship between the physical leaf position reported in the trajectory log to the radiological position derived from the EPID measurements was determined by comparing a 1.0 mm gap (projected to isocenter) between the physical leaf tips set using a feeler gauge, with the same leaf gap seen by the EPID. To verify the radiological to physical accuracy of the calibrated algorithm, two static 40 × 1.0 cm fields, one 0.5 mm mis‐calibrated and one without mis‐calibration, determined by feeler gauge, were measured and compared with the planning positions, $x_{Aj,plan}$ and $x_{Bj,plan}$, to determine $\Delta x_{\mathrm{Aj}}\left(t,\theta \right){|}_{\mathrm{dicom}}$ and $\Delta x_{\mathrm{Bj}}\left(t,\theta \right){|}_{\mathrm{dicom}}$. The measurements were also compared with positions reported in the trajectory log, $x_{Aj,traj}$ and $x_{Bj,traj}$, to determine $\Delta x_{\mathrm{Aj}}\left(t,\theta \right){|}_{\mathrm{traj}}$ and $\Delta x_{\mathrm{Bj}}\left(t,\theta \right){|}_{\mathrm{traj}}$. The comparison of the MLC positions from the trajectory log file with the measurements made using the EPID was corrected for with the measured physical — radiological leaf tip displacements. Comparisons were made to evaluate the effectiveness of the feeler gauge calibration of the trajectory log with no time delay error.

To verify the methodology for dynamic trajectory log, a set of five test patterns were delivered. Table 1 shows the summary and the descriptions of the test patterns. The mean, range, and standard deviation of the $\Delta x_{\mathrm{Aj}}\left(t,\theta \right){|}_{\mathrm{traj}}$ and $\Delta x_{\mathrm{Bj}}\left(t,\theta \right){|}_{\mathrm{traj}}$ of each plan with and without the correction were reported to assess the performance of this methodology.

**TABLE 1:** Summary of the test pattern used to verify the trajectory log QA methodology.

Plan	Description
1	40.0 × 1.0 cm static MLC
2	40.0 × 1.0 cm MLC SW traveling at 0.30 cm/s from right to left
3	40.0 × 1.0 cm MLC SW traveling at 1.25 cm/s from right to left
4	40.0 × 1.0 cm MLC SW traveling at 2.50 cm/s from right to left
5	40.0 × 1.0 cm MLC SW traveling at 2.50 cm/s from left to right
6	Clinical HN VMAT field

## 3 RESULTS

**FIG. 3:**
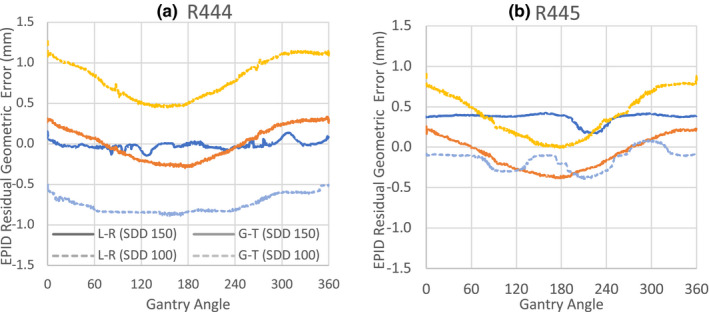
EPID residual geometric error in gun‐target (G‐T) and left–right (L‐R) direction at SSD 100 cm and SSD 150 cm of two EPID panels (a) R444 equipped with aS1200 and (b) R445 equipped with aS1000.

**FIG. 4:**
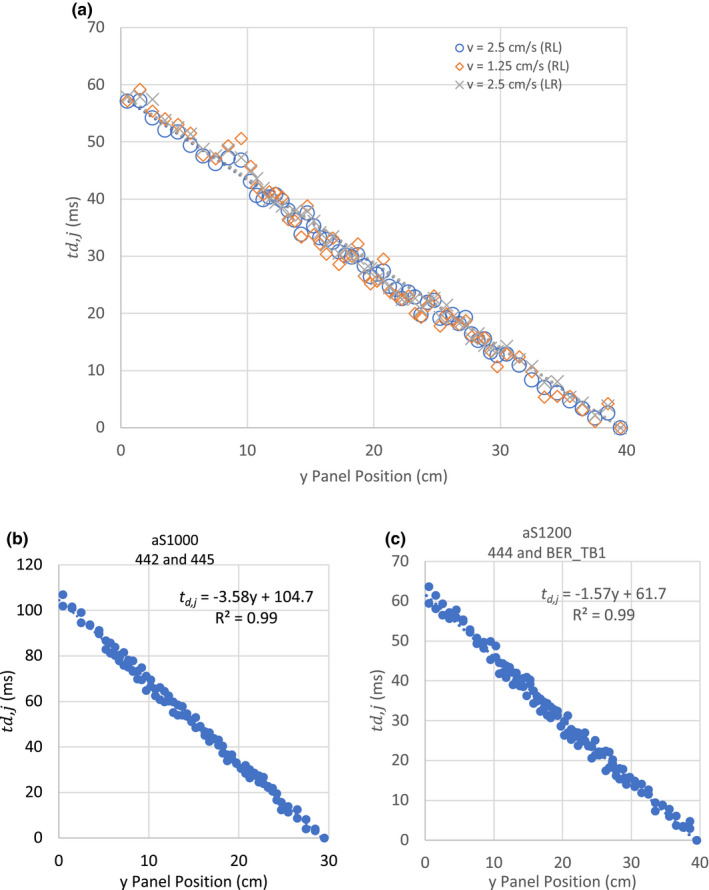
The $t_{d,j}$ variation with y panel position of (a) different MLC SW speeds, (b) aS1000 panel at 2.5 cm/s and (c) aS1200 at 2.5 cm/s.

Figure 3 shows the EPID residual geometric error in the gun‐target (G‐T) and left–right (L‐R) direction at both SDD 100 and 150 cm. At SSD 150 cm, the average error in both G‐T and L‐R were found to be 0.0 mm with maximum error of 0.3 mm. This is consistent with specification of IsoCAL. At SSD 100 cm, the average errors in G‐T and L‐R were found to be 0.75 and 0.81 mm, respectively, with the maximum error of 0.98 mm for R444. For R445, however, the G‐T and L‐R were found to be 0.40 and 0.15 with the maximum error of 0.78 mm. The $t_{d,j}$ at different y positions at different MLC speed and directions were not found to be significantly different [Fig. 4(a)]. The measured rate change in $t_{d,j}$ at different MLC travel conditions was found to be 1.47 to 1.48 ms/cm. Figure 4(b) shows the $t_{d,j}$ at different y position of two aS1000 panels at 0° collimator angle. The linear regression of the data showed a delay of 104.68 ms at 30.0 cm. The R^2^ of the regression is 0.99. Figure 4(c) shows the aS1200 results of the aS1200 panel at 0° collimator angle. The linear regression showed a delay of 61.70 ms at 40.0 cm. The R^2^ of the regression is 0.99.

**FIG. 5:**
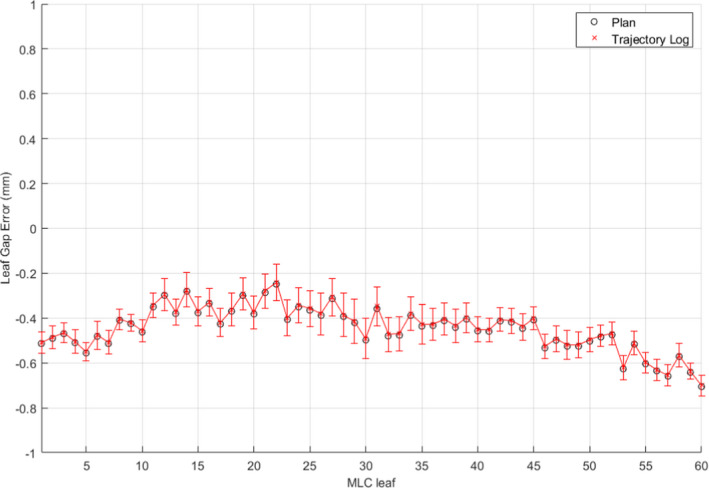
Uncorrected EPID and plan leaf gap comparison between measured leaf gap with uncorrected EPID image with plan, black circle, and trajectory log, red cross, with 0.5 mm leaf gap calibration error.

**FIG. 6:**
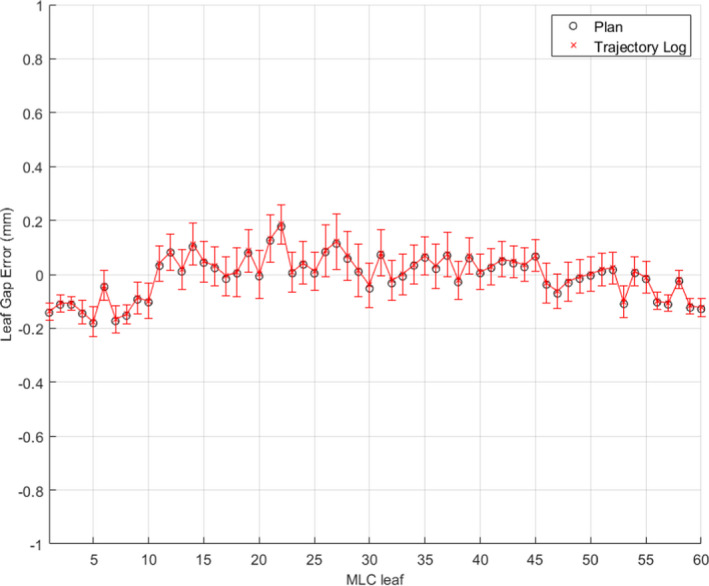
Corrected EPID and plan leaf gap comparison between measured leaf gap with corrected EPID image with plan, black circles, and trajectory log, red crosses, without leaf gap calibration error.

The radiological to physical gap correction for the M120 MLC was found to be 0.1 mm at isocenter for the EPID. With the 0.5 mm miscalibration, EPID‐based measurements showed an average of 0.5 mm gap discrepancy from the plan positions (in black circle) as shown in Fig. 5. Interestingly, the EPID‐based measurements showed the same magnitude of discrepancy with logfile‐based measurements (red cross). Figure 6 shows the measured leaf gap compared to the plan, black circles, and trajectory log, red crosses, reported gap of each leaf after the gap was adjusted to the correct position with a feeler gauge. Both the plan and trajectory gap comparisons show no significant gap error. While these results indicate that the trajectory log analysis was insensitive to the calibration error, the EPID‐based analysis was found to be sensitive to the same error.

**FIG. 7:**
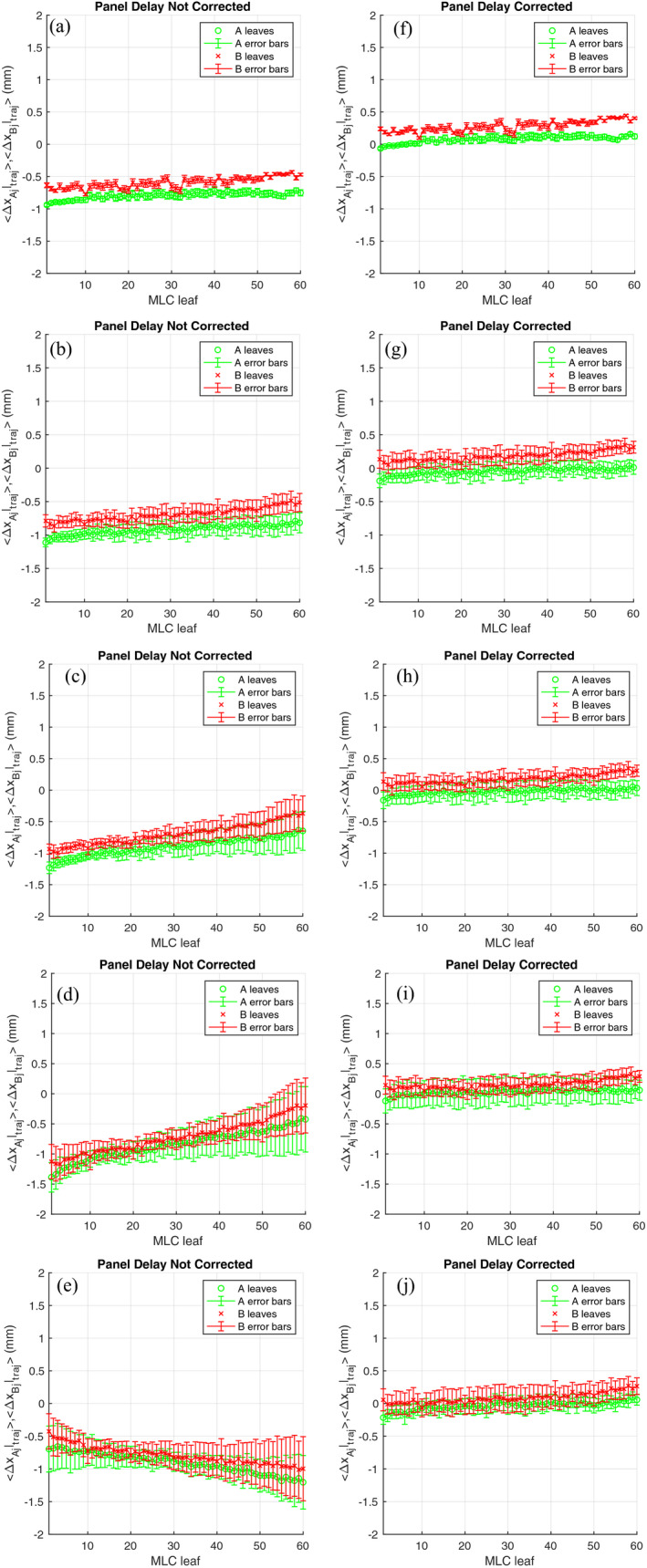
The comparison between uncorrected, (a) to (e), and corrected, (f) to (j), of the average and standard deviation of Δx_Aj_ and Δx_Bj_ for plan 1 to 5.

Using (5), the residual panel position error in the x direction (parallel to the MLC motion) was determined to be 0.84 mm at gantry 0. As this error was the same order of magnitude of the potential MLC error, the correction was deemed significant and incorporated in all the dynamic analysis. To verify the methodology for dynamic trajectory log, a set of five test patterns were delivered. Table 1 shows the summary and the descriptions of the test patterns. The mean, range, and standard deviation of the $\Delta x_{\mathrm{Aj}}\left(t,\theta \right){|}_{\mathrm{traj}}$ and $\Delta x_{\mathrm{Bj}}\left(t,\theta \right){|}_{\mathrm{traj}}$ of each plan with and without the correction were reported to assess the performance of this methodology. Figure 7 shows the comparison between the uncorrected and corrected measurements comparison with the trajectory log for plans 1 to 5. From the uncorrected measurements, the trajectory log showed an average position and centerline offset error of 0.79 and 0.69 mm, respectively, for the static aperture [Fig. 7(a)]. Correcting for the residual panel error, the average position and centerline offset error was reduced to within 0.28 and 0.18 mm (Table 1). With the SW test, similar average position and centerline errors were observed for speeds between 0.3 and 2.5 cm/s.

No significant difference in the range and standard deviation was obtained between the latency uncorrected and corrected leaf positions while they were stationary. However, while in motion, the range and standard deviation were found to increase in the uncorrected comparison with the increasing speed of the SW. The trend was observed in uncorrected comparison in Figs. 7(b) to 7(d). The skewness in the comparison was found to be directional dependent as shown in Fig. 7(e). When the speed of the field increased from 0 to 2.5 cm/s, the range of position errors increased from 0.22 to 0.92 mm and the standard deviation also increased from 0.06 to 0.54 mm (Table 2).

**TABLE 2:** Summary of the average leaf error for static and dynamic leaf motion traveling at 2.5 cm/s.

Average leaf error (mm)	A		B	
	Uncorrected	Corrected	Uncorrected	Corrected
Static				
Mean	−0.79	0.08	−0.59	0.28
Range	0.22	0.22	0.34	0.34
Max standard deviation	0.06	0.06	0.06	0.06
Dynamic (0.30 cm/s)				
Mean	−0.92	−0.05	−0.69	0.18
Range	0.32	0.22	0.38	0.29
Max standard deviation	0.16	0.15	0.16	0.16
Dynamic (1.25 cm/s)				
Mean	−0.90	−0.02	−0.70	0.18
Range	0.60	0.21	0.64	0.29
Max standard deviation	0.31	0.18	0.28	0.17
Dynamic (2.5 cm/s)				
Mean	−0.84	0.03	−0.72	0.16
Range	0.98	0.23	0.98	0.28
Max standard deviation	0.54	0.27	0.46	0.17

Applying both the panel residual and time delay correction, significant better agreement between trajectory log and measurements was observed [Figs. 7(f) to 7(j)]. The static field [Fig. 7(f)] shows a slight rotation of about 0.03° along the bank of the MLC which could be attributed to the combination of MLC systematic skewness and collimator rotation. An offset of this magnitude is unlikely to be picked up by our conventional QA tools. Similar rotation can also be observed in the corrected measurements in Figs. 7(f) to 7(j) indicating the consistency of the correction methodology with different MLC speeds. As *t_d_* is a function of distance from the center of a panel, the uncorrected positions of the MLC were found to require larger correction factors and have higher uncertainties with increasing distance from the panel center [Figs. 7(d) and 7(e)] during a dynamic delivery. Overall with the correction factors, the range of position errors was kept to within 0.05 mm from plan 1 at all speeds. About half of the magnitude of uncorrected standard deviation increase, from 0.06 to 0.27 mm, was observed for the corrected analysis (Table 2).

**FIG. 8:**
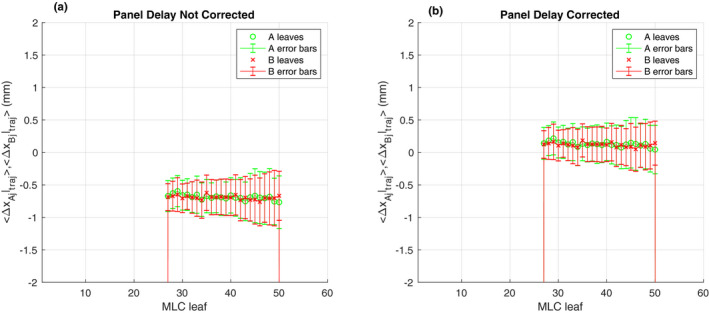
The comparison between (a) uncorrected and (b) corrected of the average and standard deviation of Δx_Aj_ and Δx_Bj_ for a clinical VMAT delivery (plan 6).

**FIG. 9:**
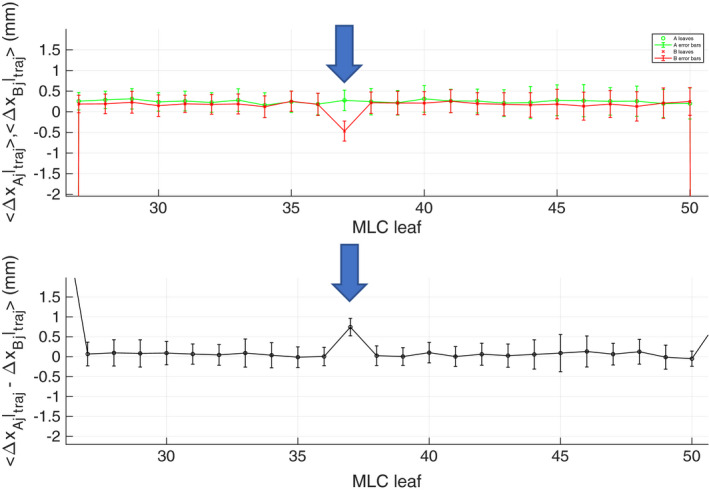
An example of a defective MLC motor during a VMAT delivery measured by EPID that was not caught by the trajectory log.

Figure 8(a) shows an average position error of −0.67 mm in the trajectory log of a VMAT delivery without correction. With *t_d_* and geometric residual corrections, however, the average position error was reduced to 0.13 mm [Fig. 8(b)]. Figure 9 shows an example the same VMAT delivery when a failing motor, B37 (blue arrow), was identified by corrected EPID image but was not identified by the trajectory log. In this case, the average position and gap error were found to be 0.6 and 0.8 mm, respectively.

## 4 DISCUSSION

In this study, we have presented a phantom‐less method to measure the dynamic MLC leaf positions with submillimeter accuracy which the conventional integrated image technique, traditionally used in portal dosimetry, cannot achieve. Similar to previous studies,^12^, ^13^ the maximum error of the EPID at SDD 150 cm was found to be within 0.5 mm. However, the maximum error was found to be in the order of 1.0 mm at SDD 100 cm which is significantly larger the error at SDD 150 cm. This can be attributed to the single SDD in the IsoCal procedure. More interestingly, the magnitude and trajectory of the flex maps were found to be machine specific. At SDD 100 cm, the precision without the machine specific panel geometric residual and temporal correction is insufficient for trajectory log verification. The results from this study showed that the readout time corrections and geometric residual corrections were able to improve the precision of both static and dynamic absolute MLC position measurements. As the method proposed by this study does not require a phantom or its setup, additional workload for clinical physicists is minimized. The accuracy reported here is comparable to the earlier work Eckhause et al,^2^ where a phantom was used to measure the position of MLC leaves.

In general, the results from the trajectory log were found to be very consistent with the measured data for both static and dynamic delivery. In this study, it was found that the log file was insensitive to MLC calibration errors. Similar to a recent study,^1^ we also found the trajectory could miss certain MLC errors. It is important to have an independent and highly accurate method of measuring static and dynamic MLC position if log files are routinely used for patient specific QA. With the increasing need of high throughput, a phantom‐less method can also improve the scalability and ease of implementation.

As this study measures the positions of MLC leaves while they are in motion, correction for the temporal delays of the EPID readout was found to be an essential correction.

## 5 CONCLUSIONS

Time delay and panel residual error correction improve EPID based MLC position measurement. These improvements provide the accuracy necessary to validate the use of trajectory logs patient specific QA.
